# Dissociation Between Acute and Late Radiation Toxicity in Locally Advanced Head and Neck Cancer: A Secondary Analysis

**DOI:** 10.7759/cureus.114400

**Published:** 2026-08-12

**Authors:** Nilesh Kucha, Tej P Soni, Dinesh Singh

**Affiliations:** 1 Radiation Oncology, Gujarat Cancer and Research Institute, Bhavnagar, IND; 2 Radiation Oncology, Bhagwan Mahaveer Cancer Hospital and Research Centre, Jaipur, IND

**Keywords:** 3d-crt, acute toxicity, chemoradiotherapy, head and neck cancer, imrt, late toxicity, secondary analysis, xerostomia

## Abstract

Background and objective

Intensity-modulated radiotherapy (IMRT) reduces both acute and late toxicity in head and neck squamous cell carcinoma (HNSCC) compared with three-dimensional conformal radiotherapy (3D-CRT). However, whether the severity of a patient’s acute treatment toxicity predicts subsequent late toxicity remains uncertain. If such an association exists, acute toxicity could identify survivors who need closer late-effect surveillance, particularly where routine dosimetric review is not feasible. Based on radiobiological differences between acute and late normal-tissue responses, we hypothesized that acute and late toxicities would be largely dissociated and prespecified this analysis to test that hypothesis. The primary objective of this study was to determine whether peak acute mucositis predicts grade ≥2 late xerostomia at 12 months. Other acute-to-late, dosimetric, and multivariable analyses were secondary or exploratory.

Methods

We reanalyzed data from 78 patients with locally advanced HNSCC treated with definitive chemoradiotherapy between 2017 and 2018. Peak acute toxicity grades, such as mucositis, dysphagia, dermatitis, and nasogastric tube requirement, were linked at the individual-patient level to 12-month late toxicity assessed using Radiation Therapy Oncology Group (RTOG)/European Organisation for Research and Treatment of Cancer (EORTC) criteria. The mean parotid dose was extracted from each treatment plan. Associations were evaluated using Fisher’s exact test and Spearman’s rank correlation, with logistic regression for exploratory analyses. A prespecified minimum detectable effect was used to assess statistical power.

Results

At 12 months, grade ≥2 late xerostomia occurred in 23.1% of IMRT patients vs 51.3% of 3D-CRT patients (p = 0.019). No acute toxicity measure significantly predicted any late endpoint; the smallest p-value was 0.142, and all CIs included the null value. However, statistical power was limited: the primary analysis had 80% power to detect only a large association (OR ≈4.1 or greater) and approximately 18% power for the observed OR of 1.69. Thus, a moderate association cannot be excluded. Patients treated with 3D-CRT had more severe acute mucositis than those treated with IMRT (43.6% vs 17.9%) and received higher mean parotid doses (35.2 vs 23.7 Gy). Although a weak correlation was observed between acute mucositis and late xerostomia (Spearman ρ = 0.23, p = 0.042), it was no longer significant after adjustment for radiotherapy technique. Both exploratory regression models failed their global significance tests and should be interpreted cautiously.

Conclusions

In this cohort, we found no convincing evidence that the severity of acute treatment toxicity predicts subsequent late toxicity, although the limited sample size means moderate associations cannot be confidently excluded. Late xerostomia was more clearly associated with radiotherapy technique and parotid dose than with the severity of the acute reaction. Acute toxicity should not be used in isolation to estimate an individual patient’s risk of late toxicity; delivered dose and treatment technique remain more informative determinants of late normal-tissue injury.

## Introduction

Head and neck squamous cell carcinoma (HNSCC) accounts for roughly a third of malignancies in Indian men, and most patients present with locoregionally advanced disease driven by tobacco and areca nut use [1,2]. Definitive radiotherapy with concurrent platinum chemotherapy is standard for these patients, and as survival has improved, more survivors live with the lasting functional costs of treatment [3].

Radiation morbidity falls into two phases. Acute toxicity, such as mucositis, painful swallowing, and skin reaction, peaks during treatment and mostly resolves. Late toxicity, such as xerostomia, fibrosis, and persistent dysphagia, appears months later and is often permanent [4]. Intensity-modulated radiotherapy (IMRT) reduces both, chiefly by sparing the parotid glands, and its salivary benefit over three-dimensional conformal radiotherapy (3D-CRT) is well established [5,6].

These two phases are not merely separated in time; they arise in tissues with different biological organizations. Acute effects occur in rapidly proliferating, hierarchically organized epithelia and are governed by dose, fraction number, and overall treatment time; late effects occur in slowly proliferating, flexibly organized parenchymal, vascular, and stromal tissues and are governed by total dose, dose per fraction, and the dose distributed across the organ [7]. Because the target cell populations and their dose-response determinants differ, radiobiological theory predicts that the severity of a patient’s acute reaction need not forecast their late injury, a prediction that has practical value only if it is tested directly in the same patients, which is what we set out to do.

Whether the two phases are linked within individual patients is less clear, and the answer has practical consequences. If a florid acute reaction reliably signaled severe late morbidity, clinicians could use it to triage survivors for intensified rehabilitation, a cheap, universally available signal valuable where dosimetric review and salivary testing are not routine. If the phases are dissociated, late risk must be judged from dose and technique instead.

The acute and late outcomes of the present cohort have been reported separately [8,9], but neither report linked them patient by patient or modeled late toxicity against measured dose. We did both. We declare our direction rather than leave it implicit: we expected dissociation on the radiobiological grounds given above and designed the analysis to test that prediction rather than to confirm an association; the central result is therefore a prespecified, mechanism-based null, and throughout we report the effect sizes the study could and could not have detected. The prespecified primary endpoint was grade ≥2 late xerostomia at 12 months, and the primary test was whether peak acute mucositis severity predicted it; the remaining 11 acute-to-late comparisons, the dosimetric analyses, and the two regression models are supporting analyses. We tested whether any acute-to-late association persisted after adjustment for radiotherapy technique and separately after adjustment for measured parotid dose, but not for both simultaneously, because dose mediates the technique effect and the two lie on one causal path.

## Materials and methods

Design and relationship to earlier reports

This is a secondary analysis of a single-institution, nonrandomized, two-arm prospective cohort treated at a tertiary cancer center in Jaipur, India. The acute toxicity and compliance outcomes were published in Cancer Treatment and Research Communications in 2020 [8]; the 12-month late toxicity outcomes were published in the International Journal of Research in Medical Sciences in 2026 [9]. No toxicity rate here is offered as a novel stand-alone finding. What is new is the patient-level linkage between the acute and late datasets, the addition of parotid dosimetry, and the regression modeling, none of which appeared in either earlier report. This overlap is stated in the cover letter and again under Additional Information, per ICMJE guidance on secondary analyses. The study is reported in accordance with the STROBE statement for observational studies.

Patients and treatment

Consecutive patients aged 18-70 years with biopsy-proven squamous cell carcinoma of the oropharynx, hypopharynx, or larynx, Karnofsky performance status ≥70 [10], and adequate organ function were enrolled between September 2017 and December 2018. We excluded patients with prior head and neck irradiation, distant metastases, non-squamous histology, or a second malignancy. All patients received 70 Gy in 35 daily 2-Gy fractions with concurrent weekly cisplatin 40 mg/m², planned on Eclipse for a Varian linear accelerator with QUANTEC dose constraints [11].

Allocation to IMRT or 3D-CRT was nonrandomized. Consecutive eligible patients were enrolled as they presented, and the radiotherapy technique for each was decided at the discretion of the treating senior consultants (the authors), weighing the substantially greater planning and delivery time that IMRT demands, together with the associated logistic and cost considerations, against the individual patient’s circumstances. This reflects routine real-world practice at an Indian tertiary center during 2017-2018 and is consistent with the nonrandomized design reported in the parent studies [8,9].

Baseline demographic and clinicopathological characteristics by arm are shown in the Results, reproduced from the late-toxicity parent report [9], and cross-checked against the study dataset. The arms did not differ significantly in age (58.6 vs 58.9 years, p = 0.887), sex (79.5% male in both, p = 1.000), Karnofsky performance status (median 90 in both, p = 0.161), primary subsite (oropharynx 48/78, larynx 19/78, hypopharynx 11/78; p = 0.340), T stage (p = 0.519), or N stage (p = 0.088). The cohort is therefore balanced on all measured baseline factors, which substantiates the balance previously asserted only for age. The subsite remains worth flagging despite the balance because laryngeal primaries (more frequent in the 3D-CRT arm, 12 vs 7) receive low parotid doses regardless of technique.

**Figure 1:**
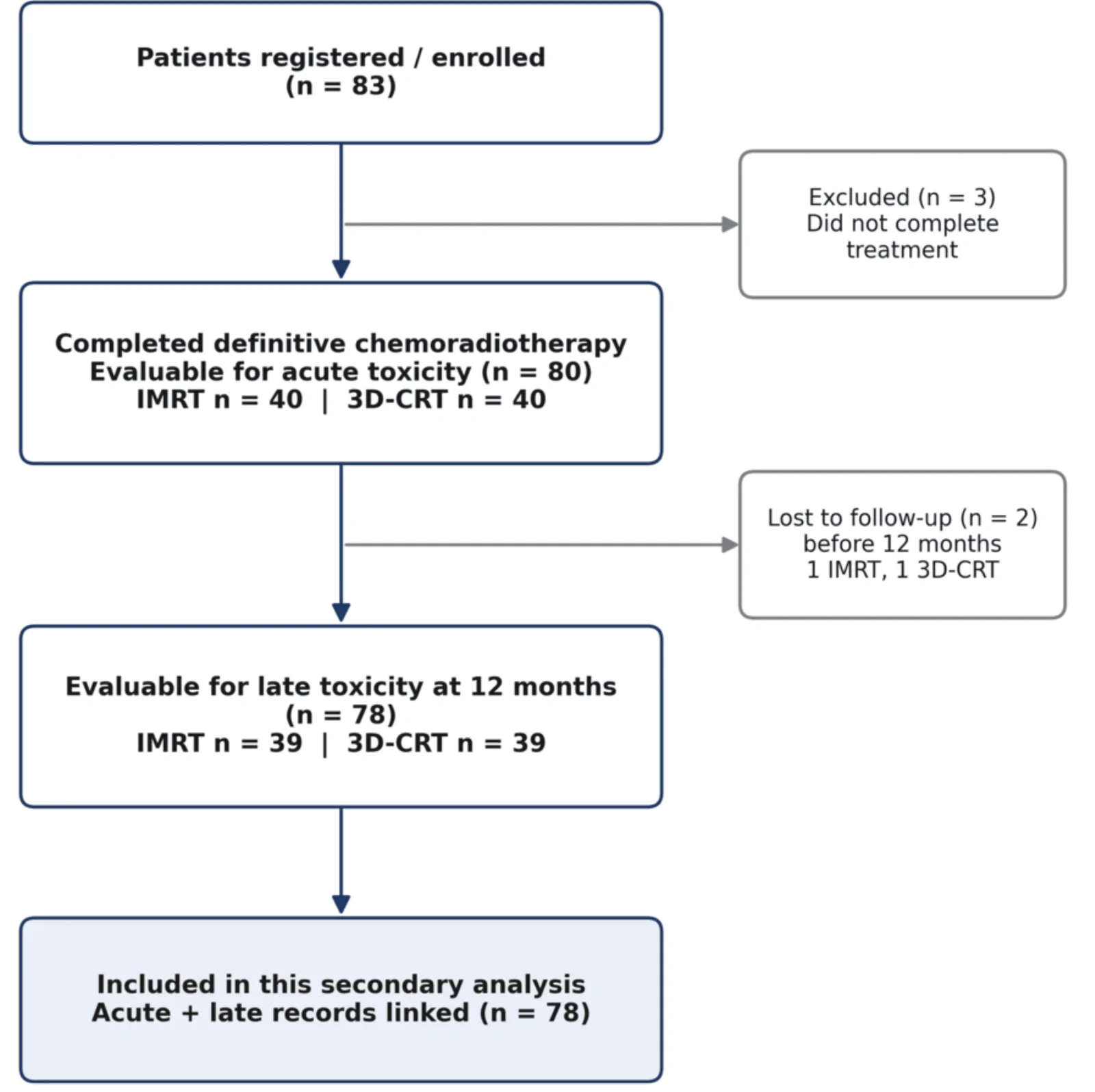
Patient flow through the study cohort. Eighty-three patients were enrolled between September 2017 and December 2018. Three patients did not complete definitive chemoradiotherapy and were excluded from analysis. Eighty patients completed treatment and were evaluable for acute toxicity (40 IMRT and 40 3D-CRT). During follow-up, one patient in each treatment arm was lost before the 12-month assessment, leaving 78 patients (39 IMRT and 39 3D-CRT) evaluable for late toxicity and included in the present secondary analysis. 3D-CRT, three-dimensional conformal radiotherapy; IMRT, intensity-modulated radiotherapy

Patient flow was as follows. Eighty-three patients were registered; three did not complete treatment and were excluded, leaving 80 who completed definitive chemoradiotherapy and were evaluable for acute toxicity (40 IMRT, 40 3D-CRT), the cohort of the acute-toxicity parent report [8]. Between treatment completion and the 12-month assessment, one patient in each arm was lost to follow-up, leaving 78 patients evaluable for late toxicity (39 IMRT and 39 3D-CRT), the cohort of the late-toxicity parent report [9] and of the present secondary analysis. The apparent difference between the 80 (40 + 40) of [8] and the 78 (39 + 39) of [9] is therefore fully explained by these two losses to follow-up, one per arm; the 100% treatment-completion rate reported in [9] refers to the analysis-stage cohort, all of whom completed treatment. No patient was excluded for locoregional recurrence or death before the 12-month assessment, so late toxicity was not confounded by recurrent disease masquerading as radiation injury. The full flow is shown in Figure 1.

Toxicity and dosimetric data

Acute toxicity was graded weekly during treatment using NCI-CTCAE v4.03 [12]. For this analysis, we derived each patient’s peak acute grade across all treatment weeks for mucositis, dysphagia, and dermatitis and recorded nasogastric tube requirement as a binary variable. Late toxicity was graded at 12 months using Radiation Therapy Oncology Group (RTOG)/European Organisation for Research and Treatment of Cancer (EORTC) criteria [13] for xerostomia, dysphagia, and submucosal fibrosis, with grade ≥2 prespecified as clinically significant. Acute and late records were linked by registration number.

Late toxicity was graded at 12 months by the treating clinician using RTOG/EORTC criteria [9,13] and was therefore not blinded to treatment arm; no independent interobserver agreement statistic, objective sialometry, or patient-reported outcome was recorded. Because RTOG/EORTC late xerostomia grading is clinician-assessed and subjective, an unblinded assessor expecting dry mouth after 3D-CRT is a plausible source of differential misclassification in the direction reported; this is addressed in the Limitations.

The nasogastric tube requirement was defined as reactive placement, a tube inserted when a patient could no longer maintain adequate oral intake during treatment, not prophylactic placement; no patient received a prophylactic tube. It is therefore a toxicity-driven outcome rather than an institutional-policy variable, which supports its use as a severity measure.

The mean dose to each parotid gland was extracted from the approved plan; the lower-dose gland was used as the primary dosimetric exposure since it determines residual function. The 26-Gy dichotomization threshold was prespecified on the basis of a literature review and the mean parotid dose constraint used in the parent study’s planning protocol [8]; it was not selected after inspecting the dose distribution in this cohort, so the cut point was fixed a priori rather than optimized against the outcome. This 26-Gy threshold is more permissive than the QUANTEC recommendation, which advises a mean dose below approximately 20 Gy for at least one gland or below 25 Gy for both [14]; we report the QUANTEC figures as the stricter comparator.

Statistical analysis

We compared categorical variables with two-sided Fisher’s exact tests, applied uniformly to all 2 × 2 tables. The uncorrected Pearson χ² (one degree of freedom) is reported alongside each comparison; expected counts fell below five only for the acute dermatitis comparisons. For the primary xerostomia endpoint, we also report the Yates-corrected χ² for direct comparison with the parent publication [9]. ORs carry 95% CIs by the Woolf logit method; relative risk is given where a risk interpretation applies. Ordinal associations used Spearman correlation, and continuous variables were analyzed using the independent-samples t-test, reported with the test statistic, degrees of freedom, and Cohen’s d.

Radiotherapy technique and mean parotid dose were not entered into one model as competing predictors. This was not on grounds of collinearity, as the variance inflation factor of 2.51 is well within acceptable limits, but because the mean parotid dose lies on the causal pathway between technique and late xerostomia. Adjusting a total technique effect for its own mediator biases the estimate. We therefore fitted two parallel logistic models for grade ≥2 late xerostomia: Model A containing technique and Model B substituting measured lower parotid dose; both also contained acute mucositis grade and age. With 29 events, we limited each model to three covariates (9.7 events per variable) and reported coefficients, standard errors, z statistics, ORs, likelihood-ratio χ², McFadden pseudo-R², and area under the receiver operating characteristic curve.

Because the principal hypothesis is a null, we report the study’s sensitivity. For the primary acute-to-late test, such as acute mucositis grade 3 (n = 24) vs ≤grade 2 (n = 54) against grade ≥2 late xerostomia, reference rate 33.3%, the minimum OR detectable with 80% power at two-sided α = 0.05 was approximately 4.1 (relative risk ≈2.0); the study had only about 18% power to detect the observed OR of 1.69. All null acute-to-late findings are interpreted within these bounds.

Acute mucositis was analyzed at the prespecified grade 3 threshold, defined as severe, confluent mucositis, throughout; this was the clinically significant cut point identified a priori. We did not additionally model mucositis as a continuous ordinal grade. We note that the single nominally significant univariate association (Spearman ρ = +0.23, p = 0.042) used ordinal coding, whereas the adjusted models used the dichotomy, and we consider the implications of this coding choice in the Limitations.

We applied no multiplicity correction to the 12 exploratory acute-to-late tests because none approached nominal significance (smallest p = 0.142). Significance was two-sided (p < 0.05). Analyses used Python 3.12 with SciPy 1.17 and statsmodels 0.14.

## Results

Cohort and toxicity frequencies

The arms did not differ significantly on any measured baseline characteristic, such as age, sex, KPS, subsite, T stage, or N stage (all p > 0.05; Table 1, from the parent report [9]). Peak acute mucositis reached grade 3 in 24 patients (30.8%), acute dysphagia grade ≥2 in 57 (73.1%), and acute dermatitis grade ≥2 in 69 (88.5%); 34 patients (43.6%) required a nasogastric tube. At 12 months, grade ≥2 xerostomia was present in 29 patients (37.2%), dysphagia in 49 (62.8%), and submucosal fibrosis in 29 (37.2%).

**Table 1:** Baseline demographic and clinical characteristics by treatment arm. Baseline demographic and clinicopathological characteristics were reproduced from the late-toxicity parent report [9] and cross-checked against the study dataset. The arms did not differ significantly on any characteristic, such as age, sex, KPS, primary subsite, T stage, or N stage (all p > 0.05), so the cohort is balanced on measured baseline factors. 3D-CRT, three-dimensional conformal radiotherapy; IMRT, intensity-modulated radiotherapy; KPS, Karnofsky performance status

Characteristic	IMRT (n = 39)	3D-CRT (n = 39)	p-value
Age, years (mean ± SD)	58.6 ± 9.2	58.9 ± 8.3	0.887
Sex: male	31 (79.5%)	31 (79.5%)	1.000
Sex: female	8 (20.5%)	8 (20.5%)	-
KPS, median	90	90	0.161
Subsite: oropharynx	27 (69.2%)	21 (53.8%)	0.340
Subsite: hypopharynx	5 (12.8%)	6 (15.4%)	-
Subsite: larynx	7 (17.9%)	12 (30.8%)	-
T stage: T2	3 (7.7%)	5 (12.8%)	0.519
T stage: T3	18 (46.2%)	21 (53.8%)	-
T stage: T4a	17 (43.6%)	13 (33.3%)	-
T stage: T4b	1 (2.6%)	0 (0.0%)	-
N stage: N1	1 (2.6%)	3 (7.7%)	0.088
N stage: N2	24 (61.5%)	30 (76.9%)	-
N stage: N3	14 (35.9%)	6 (15.4%)	-

Table 2 gives the distribution of acute and late toxicity by technique. Severe acute mucositis and nasogastric tube requirement were both significantly more frequent with 3D-CRT.

**Table 2:** Peak acute toxicity and grade ≥2 late toxicity by radiotherapy technique (n = 78). ORs are expressed with IMRT as the exposed group, so an OR below 1 favors IMRT. χ² values are uncorrected Pearson statistics reported for transparency; p values are two-sided Fisher’s exact tests, applied uniformly across all 2 × 2 comparisons. For the primary xerostomia endpoint, the Yates-corrected χ² was 5.49 (p = 0.019). Severe acute mucositis and nasogastric tube requirement were significantly more frequent with 3D-CRT, whereas acute dysphagia and dermatitis did not differ between arms. Acute grades are per-patient peak values across treatment weeks (NCI-CTCAE v4.03); late grades were scored at 12 months (RTOG/EORTC). 3D-CRT, three-dimensional conformal radiotherapy; EORTC, European Organisation for Research and Treatment of Cancer; IMRT, intensity-modulated radiotherapy; RTOG, Radiation Therapy Oncology Group

Toxicity endpoint	IMRT (n = 39)	3D-CRT (n = 39)	χ² (df = 1)	OR (95% CI)	p-value
Peak acute mucositis grade 3	7 (17.9%)	17 (43.6%)	6.02	0.28 (0.10-0.80)	0.026
Peak acute dysphagia ≥grade 2	30 (76.9%)	27 (69.2%)	0.59	1.48 (0.54-4.06)	0.610
Peak acute dermatitis ≥grade 2	35 (89.7%)	34 (87.2%)	0.13	1.29 (0.32-5.20)	1.000
Nasogastric tube required	10 (25.6%)	24 (61.5%)	10.22	0.22 (0.08-0.57)	0.003
Late xerostomia ≥grade 2	9 (23.1%)	20 (51.3%)	6.64	0.28 (0.11-0.76)	0.018
Late dysphagia ≥grade 2	24 (61.5%)	25 (64.1%)	0.05	0.90 (0.36-2.25)	1.000
Late submucosal fibrosis ≥grade 2	12 (30.8%)	17 (43.6%)	1.37	0.58 (0.23-1.46)	0.349

Late toxicity was not predicted by acute severity within the limits of statistical power

Late toxicity rates appeared mostly uniform when stratified by the severity of the acute mucosal reaction. Specifically, grade ≥2 late xerostomia affected 45.8% of patients who experienced grade 3 acute mucositis compared with 33.3% of those with grade ≤2 (p = 0.319); late dysphagia was observed in 62.5% vs 63.0% (p = 1.000). No statistically significant association was established between any of the four acute parameters and the late outcomes. Across all 12 calculated comparisons, the lowest unadjusted p-value was 0.142, and the CIs for every OR encompassed unity (Table 3). It is critical to recognize these findings as null results within an underpowered clinical framework rather than definitive proof of zero effect. For the primary analysis, this study had 80% power to identify only an OR of ~4.1 or greater, and it retained only ~18% power to detect the observed OR of 1.69. Consequently, potential associations as high as roughly fourfold cannot be completely ruled out. Measured Spearman correlations remained consistently weak across all evaluations; the sole relationship reaching nominal significance was acute mucositis vs late xerostomia (ρ = +0.23, p = 0.042, n = 78), an observation detailed further below.

**Table 3:** Association between each acute toxicity measure and each grade ≥2 late endpoint (n = 78). All 12 acute-to-late comparisons were nonsignificant; the final row shows the dosimetric comparison for contrast. χ² values are uncorrected Pearson statistics; p values are two-sided Fisher’s exact tests. The smallest acute-to-late p-value was 0.142, so multiplicity adjustment would not alter the conclusions. Given the minimum detectable OR of ≈4.1, these null findings do not exclude moderate associations.

Acute measure	Late endpoint	Rate if present vs absent	OR (95% CI)	χ² (df = 1)	p-value
Acute mucositis grade 3	Xerostomia	45.8% vs 33.3%	1.69 (0.63-4.52)	1.11	0.319
Acute mucositis grade 3	Dysphagia	62.5% vs 63.0%	0.98 (0.36-2.65)	0.00	1.000
Acute mucositis grade 3	Submucosal fibrosis	41.7% vs 35.2%	1.32 (0.49-3.52)	0.30	0.619
Acute dysphagia ≥grade 2	Xerostomia	40.4% vs 28.6%	1.69 (0.57-5.00)	0.91	0.432
Acute dysphagia ≥grade 2	Dysphagia	59.6% vs 71.4%	0.59 (0.20-1.75)	0.91	0.432
Acute dysphagia ≥grade 2	Submucosal fibrosis	40.4% vs 28.6%	1.69 (0.57-5.00)	0.91	0.432
Acute dermatitis ≥grade 2	Xerostomia	39.1% vs 22.2%	2.25 (0.43-11.65)	0.97	0.471
Acute dermatitis ≥grade 2	Dysphagia	62.3% vs 66.7%	0.83 (0.19-3.59)	0.06	1.000
Acute dermatitis ≥grade 2	Submucosal fibrosis	40.6% vs 11.1%	5.46 (0.65-46.14)	2.96	0.142
Nasogastric tube required	Xerostomia	44.1% vs 31.8%	1.69 (0.67-4.28)	1.24	0.346
Nasogastric tube required	Dysphagia	61.8% vs 63.6%	0.92 (0.37-2.33)	0.03	1.000
Nasogastric tube required	Submucosal fibrosis	32.4% vs 40.9%	0.69 (0.27-1.76)	0.60	0.486
Lower parotid Dmean <26 Gy vs ≥26 Gy	Xerostomia	21.9% vs 47.8%	0.31 (0.11-0.85)	5.44	0.031

Late xerostomia tracked technique

Technique was the strongest measured correlate of late xerostomia: 23.1% with IMRT vs 51.3% with 3D-CRT (Yates-corrected χ² = 5.49, df = 1, p = 0.019; Fisher’s exact p = 0.018; uncorrected χ² = 6.64; relative risk 0.45, 95% CI 0.24-0.86; OR 0.29, 95% CI 0.11-0.76). Severe acute mucositis was also more common with 3D-CRT (43.6% vs 17.9%, p = 0.026), so technique was associated with both the acute reaction and the late salivary outcome, which makes the weak acute mucositis correlation potentially confounded rather than causal.

**Figure 2:**
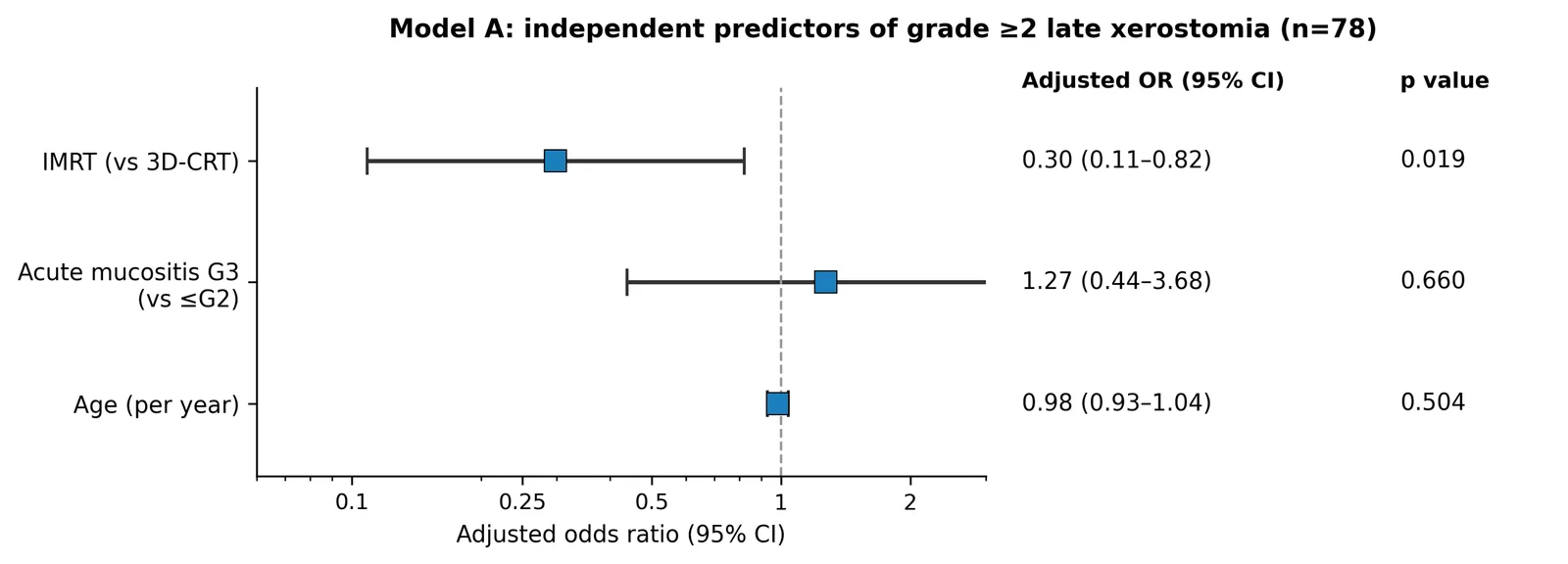
Independent predictors of late xerostomia (Model A). Forest plot of the multivariable logistic regression model containing radiotherapy technique, peak acute mucositis grade, and age. IMRT was the only independent predictor of grade ≥2 late xerostomia; peak acute mucositis grade 3 was not associated with the outcome after adjustment. Squares mark adjusted ORs, horizontal lines indicate the 95% CIs, and the dashed line indicates an OR of 1; the accompanying column lists the same estimates numerically. IMRT, intensity-modulated radiotherapy

Model A was consistent with this. IMRT remained associated with lower odds of late xerostomia (adjusted OR 0.30, 95% CI 0.11-0.82, p = 0.019), while acute mucositis grade 3 was unrelated once technique was accounted for (adjusted OR 1.27, 95% CI 0.44-3.68, p = 0.660); age was also unrelated (Table 4, Figure 2). The model as a whole did not reach significance (likelihood-ratio p = 0.062), and discrimination was modest (area under the curve (AUC) = 0.674), so these coefficients are exploratory; the disappearance of the acute mucositis signal on adjustment is consistent with the univariate correlation reflecting technique rather than an acute-to-late causal pathway and is being rechecked with ordinal coding (Methods).

**Table 4:** Multivariable logistic regression for grade ≥2 late xerostomia - Model A (radiotherapy technique). Model likelihood-ratio χ² = 7.35 (df = 3), p = 0.062; McFadden pseudo-R² = 0.071; AUC = 0.674; 78 patients, 29 events (9.7 events/variable). The overall model did not reach significance, and discrimination was modest; the model is exploratory. AUC, area under the curve; IMRT, intensity-modulated radiotherapy

Variable	β	SE	z	Adjusted OR (95% CI)	p-value
IMRT (vs 3D-CRT)	-1.209	0.516	-2.35	0.30 (0.11-0.82)	0.019
Acute mucositis grade 3 (vs ≤grade 2)	0.239	0.543	0.44	1.27 (0.44-3.68)	0.660
Age (per year)	-0.019	0.029	-0.67	0.98 (0.93-1.04)	0.504

Parotid dose explained the technique effect but nearly repeated the technique analysis

**Figure 3:**
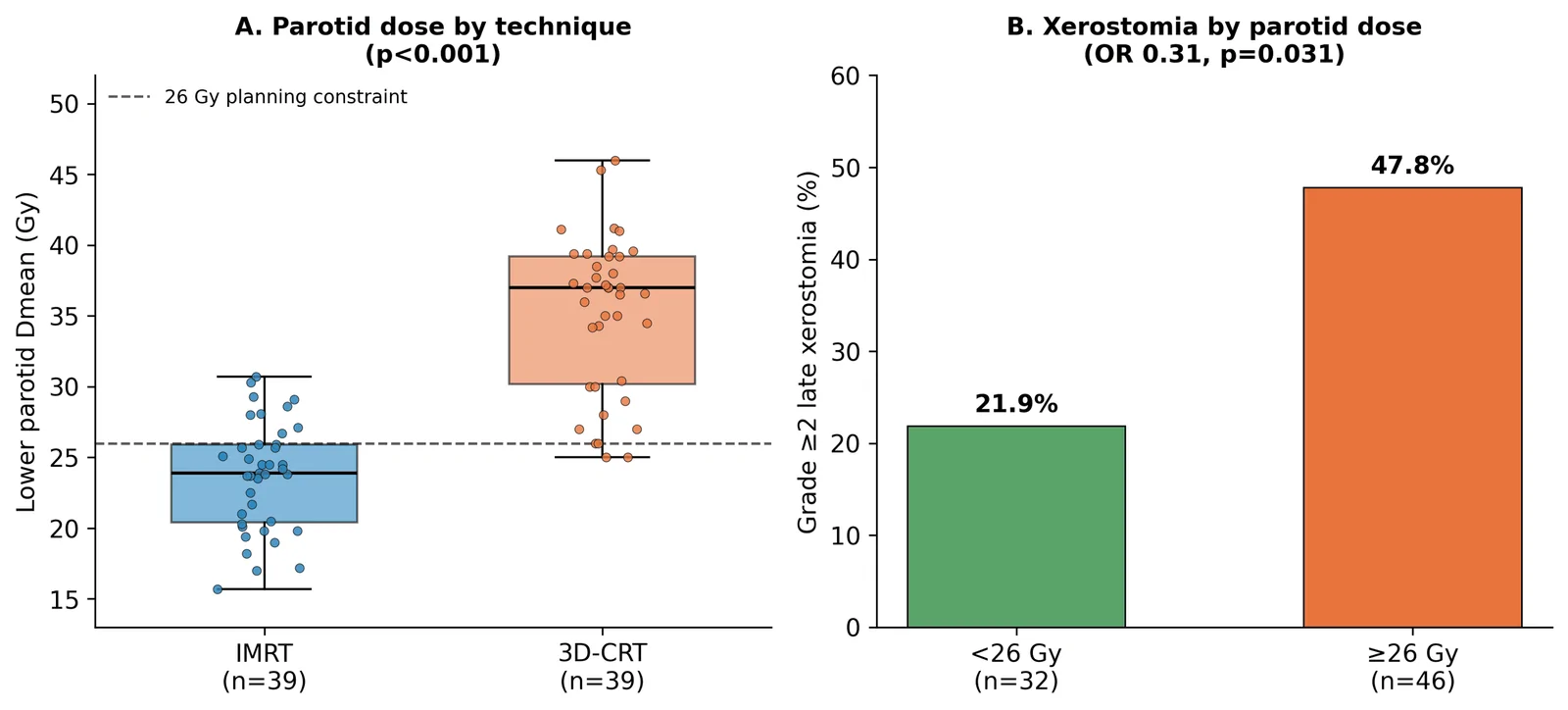
Dosimetric basis of the technique effect. (A) Mean lower-parotid dose by radiotherapy technique. IMRT delivered a substantially lower parotid dose than 3D-CRT (23.7 ± 3.8 Gy vs 35.2 ± 5.5 Gy; t = -10.71, df = 76, p < 0.001). Boxes show the median and interquartile range, individual points show each patient, and the dashed line marks the 26-Gy parotid constraint. (B) Grade ≥2 late xerostomia by parotid dose stratum. Patients whose lower parotid received less than 26 Gy had less than half the rate of clinically significant late xerostomia (21.9% vs 47.8%; OR 0.31, 95% CI 0.11-0.85; Fisher’s exact p = 0.031). 3D-CRT, three-dimensional conformal radiotherapy; IMRT, intensity-modulated radiotherapy

Dosimetry localized the mechanism. The mean lower-parotid dose was far lower with IMRT than 3D-CRT (23.7 ± 3.8 vs 35.2 ± 5.5 Gy; t = -10.71, df = 76, p < 0.001, Cohen’s d = -2.42; Table 5, Figure 3), confirming that the techniques differ in the dose reaching the salivary glands. Dose in turn tracked outcome: lower-parotid dose was higher in patients who developed grade ≥2 late xerostomia (31.9 ± 7.1 vs 28.0 ± 7.4 Gy; t = 2.28, df = 76, p = 0.026, Cohen’s d = +0.53) and correlated with late xerostomia grade (ρ = +0.27, p = 0.017, n = 78). Dichotomized at 26 Gy, patients whose lower parotid received under 26 Gy had less than half the rate of clinically significant late xerostomia (21.9% vs 47.8%; OR 0.31, 95% CI 0.11-0.85; Fisher’s exact p = 0.031).

**Table 5:** Comparison of continuous variables (independent-samples t-test). Group 1 and Group 2 correspond to the order stated in the comparison label. Cohen’s d values of 0.2, 0.5, and 0.8 denote small, moderate, and large effects, respectively. 3D-CRT, three-dimensional conformal radiotherapy; IMRT, intensity-modulated radiotherapy

Comparison	Group 1 (mean ± SD)	Group 2 (mean ± SD)	t	df	p-value	Cohen’s d
Lower parotid Dmean by technique (IMRT vs 3D-CRT)	23.7 ± 3.8	35.2 ± 5.5	-10.71	76	<0.001	-2.42
Lower parotid Dmean by late xerostomia (present vs absent)	31.9 ± 7.1	28.0 ± 7.4	2.28	76	0.026	0.53
Age by technique (IMRT vs 3D-CRT)	58.6 ± 9.2	58.9 ± 8.3	-0.14	76	0.887	-0.03

This dose stratification is, however, very nearly the technique comparison performed a second time. The cross-tabulation (Table 6) makes this explicit: the <26-Gy stratum (n = 32) is composed entirely of IMRT patients, and the ≥26-Gy stratum (n = 46) comprises all 39 3D-CRT patients plus 7 IMRT patients. The dose finding, therefore, corroborates little beyond Table 2, it is the same 78 patients split almost identically and analyzed again, not an independent line of evidence. We present it as a mechanistic illustration, not as confirmation.

**Table 6:** Cross-tabulation of technique against lower-parotid dose stratum. The dose stratum is almost collinear with technique. The per-cell xerostomia event split (seven of the nine IMRT events in the <26-Gy stratum and two among the nine IMRT patients ≥26 Gy) is consistent with the verified technique-level event totals (nine IMRT, 20 3D-CRT, 29 total); the dose-stratum patient counts derive from the treatment-planning dosimetry. 3D-CRT, three-dimensional conformal radiotherapy; IMRT, intensity-modulated radiotherapy

Radiotherapy technique	<26 Gy	≥26 Gy	Total
IMRT	32	7	39
3D-CRT	0	39	39
Total	32	46	78
Grade ≥2 xerostomia events	7 (21.9%)	22 (47.8%)	29

**Figure 4:**
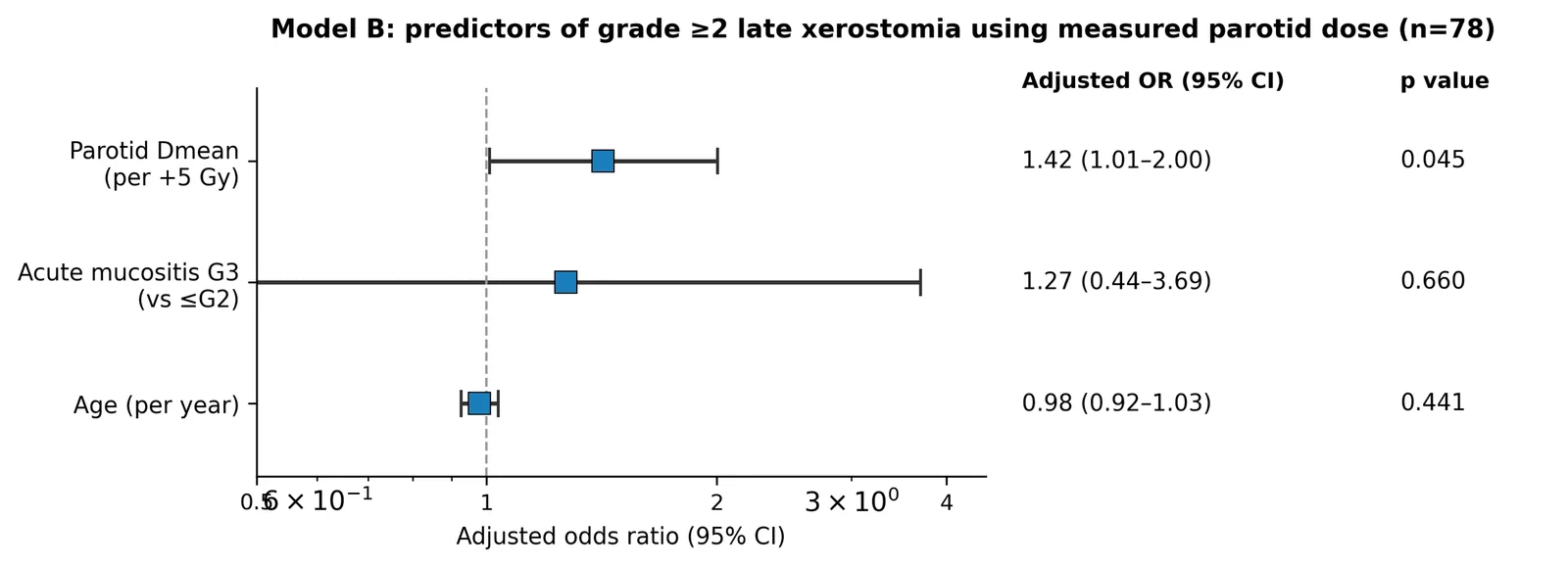
Predictors of late xerostomia using measured parotid dose (Model B). Model B: multivariable logistic regression for grade ≥2 late xerostomia with radiotherapy technique replaced by measured lower-parotid mean dose. Squares mark adjusted ORs, horizontal lines indicate the 95% CIs, and the dashed line indicates an OR of 1; the accompanying column lists the same estimates numerically. Higher parotid dose predicted late xerostomia; acute mucositis grade did not.

In Model B, replacing technique with measured dose, the higher parotid dose was associated with late xerostomia (adjusted OR 1.42 per additional 5 Gy, 95% CI 1.01-2.00, p = 0.045), while acute mucositis again was not (adjusted OR 1.27, p = 0.660) (Table 7, Figure 4). Technique and parotid dose are strongly correlated (r = -0.78), so entering both attenuates each, and neither retains significance, the expected behavior when dose lies on the causal path between technique and outcome. The data are thus consistent with parotid dose mediating the technique effect, although formal mediation cannot be established at this sample size. Model B also failed its global test (likelihood-ratio p = 0.123, AUC = 0.677), and the dose coefficient’s lower confidence bound of 1.01 at p = 0.045 is fragile; this estimate is exploratory.

**Table 7:** Multivariable logistic regression for grade ≥2 late xerostomia - Model B (measured parotid dose). Model likelihood-ratio χ² = 5.77 (df = 3), p = 0.123; McFadden pseudo-R² = 0.056; AUC = 0.677. Technique and parotid dose were strongly correlated (r = -0.78; variance inflation factor = 2.51 each) and were not entered together as primary predictors because dose mediates the technique effect; the model is exploratory. AUC, area under the curve

Variable	β	SE	z	Adjusted OR (95% CI)	p-value
Lower parotid Dmean (per +5 Gy)	0.351	0.175	2	1.42 (1.01-2.00)	0.045
Acute mucositis grade 3 (vs ≤grade 2)	0.239	0.545	0.44	1.27 (0.44-3.69)	0.660
Age (per year)	-0.022	0.029	-0.77	0.98 (0.92-1.03)	0.441

## Discussion

This analysis linked the acute and late toxicity records of a prospectively followed HNSCC cohort patient by patient, something neither earlier report attempted. We found no evidence that the severity of a patient’s reaction during treatment predicted their grade ≥2 xerostomia, dysphagia, or fibrosis a year later. This represents a failure to demonstrate an association rather than proof of its absence: with 78 patients and 29 events, the study could detect only ORs of roughly 4 or above for the primary endpoint, so acute-to-late associations up to about fourfold remain compatible with our data. The one apparent link, acute mucositis with late xerostomia, did not survive adjustment because 3D-CRT independently produced both a worse acute reaction and a higher parotid dose.

Radiobiological basis for the dissociation

The independence we observed is what classical radiobiology predicts, and because the reviewers rightly asked for it, the mechanism is worth setting out in full. Normal-tissue reactions are conventionally divided into early- and late-responding types according to the proliferative organization of the tissue and its sensitivity to fraction size [7]. The two classes have different target cells, different latencies, and different dose-response determinants, and the acute and late toxicities measured here fall cleanly into the two classes.

Mechanism of the Early Sequelae

Acute mucositis, acute dysphagia, and dermatitis are early effects arising in hierarchically organized epithelia, oral and pharyngeal mucosa, and epidermis, in which a small compartment of basal stem cells feeds a larger transit-amplifying compartment that matures into the functional surface layer. Fractionated irradiation sterilizes dividing basal clonogens; the surface denudes when cell loss outpaces production, producing confluent mucositis at cumulative doses around 30-50 Gy, and the tissue heals once surviving clonogens repopulate. Early-responding epithelia have a high α/β ratio (of the order of 10 Gy) and, being rapidly proliferating, are strongly influenced by overall treatment time, including the accelerated repopulation that begins during the third to fourth week of a conventional course [7]. The severity and timing of the acute reaction are therefore set by total dose, fraction number, treatment time, and the volume of mucosa in the high-dose region, quantities that are largely spent and resolved by the time late injury declares itself.

Mechanism of the Late Sequelae

Late xerostomia, submucosal fibrosis, and persistent late dysphagia are late effects arising in flexibly organized, slowly proliferating tissues, where injury reflects the combined loss of parenchymal cells, microvascular endothelium, and fibroblast/stromal elements rather than acute epithelial denudation. Late-responding tissues have a low α/β ratio (roughly 3 Gy) and are therefore sensitive to dose per fraction but relatively insensitive to overall treatment time, and they express a dose-volume dependence captured by the functional-subunit concept and by QUANTEC mean-dose constraints [11,14]. For the parotid specifically, the serous acinar cell is the critical target: early secretory and membrane dysfunction is followed by largely irreversible acinar loss when radiation-damaged stem/progenitor cells fail to regenerate acini, compounded by microvascular and stromal injury [15]. The consequence is that late salivary function is governed by the mean dose delivered to the gland, with partial recovery over one to two years when that dose is low, and is essentially decoupled from the kinetics of the mucosal reaction.

Radiobiological Explanation of the Dissociation

Because the early and late endpoints arise in different tissue compartments, with different target cells, different α/β ratios, and different governing dose metrics, there is no mechanistic pathway by which the intensity of a patient’s mucosal reaction should forecast their acinar loss. This separation is sharpened by the design of the parent cohort: every patient received an identical schedule, 70 Gy in 35 fractions of 2 Gy with concurrent weekly cisplatin, so the fractionation and time variables that drive the acute reaction were held constant across the series. The only late-determining quantity that varied between patients was therefore the physical dose distribution, in particular the mean parotid dose, which is set by beam geometry and inverse planning and is unrelated to how briskly the mucosa reacted. Our data are the clinical expression of this: 3D-CRT delivered about 12 Gy more to the lower parotid, and it was that dose difference, not the acute reaction, that best explained the late salivary outcome. The early-vs-late tissue framework thus predicts dissociation for xerostomia, and dissociation is what we found.

The Exception: Consequential Late Effects

There is one well-recognized situation in which acute and late injury are genuinely linked, and it is worth stating so that our conclusion is not overgeneralized. In consequential late effects, a severe and prolonged acute epithelial reaction breaches the mucosal or cutaneous barrier deeply enough to injure the underlying connective tissue and vasculature so that the acute reaction itself precipitates late fibrosis, ulceration, or necrosis; here acute severity does predict late morbidity [16]. Xerostomia is not a consequential effect; acinar loss does not arise from the mucosal reaction, so dissociation is expected and observed. A consequential link is biologically more plausible for late submucosal fibrosis or persistent dysphagia, where severe confluent mucositis with delayed healing could in principle drive later fibrosis; we saw no such association for either endpoint, but with 29 and 49 events, respectively, and the wide CIs reported above, the study was underpowered to exclude a modest consequential contribution. This nuance qualifies, rather than overturns, the main message: for the dose-driven late effect that dominates salivary morbidity, acute severity carries no useful predictive information.

Clinical implications

The clinical implication is direct, particularly for Indian district and regional centers still delivering 3D-CRT. A florid acute reaction should not be read as a warning of severe late morbidity, and a mild acute course should not reassure, with the caveat that our study could not exclude a moderate acute-to-late association, so this guidance rests as much on the radiobiology above as on the statistics. Select survivors for salivary rehabilitation and surveillance on the basis of delivered dose, specifically whether the better-spared parotid stayed under the 26-Gy constraint,or the still lower QUANTEC figures, rather than on how the patient looked during treatment. Expanding IMRT capacity and auditing parotid dose where IMRT is already available remain the effective levers, consistent with PARSPORT, with Gupta et al.’s Indian randomized data, and with meta-analyzed evidence [5,6,17]. Because this is a nonrandomized cohort with no adjustment for subsite or stage, we frame these as associations and recommendations, not as established causal effects.

Neither late dysphagia nor late fibrosis showed a technique or acute-reaction association. Late dysphagia was high in both arms (above 60%), reflecting the predominance of oropharyngeal primaries (48 of 78, 61.5%) and the absence of dysphagia-optimized planning; without explicit constraints on the pharyngeal constrictors and supraglottic larynx, IMRT confers little swallowing benefit [18,19]. We deliberately did not analyze constrictor dose as a sparing metric, since in laryngeal and hypopharyngeal primaries the constrictor lies within the target volume and cannot be spared. The lower fibrosis rate with IMRT did not reach significance, most likely because of limited statistical power.

Limitations

This study has several important limitations that should be considered when interpreting its findings.

First, the study was underpowered to confidently exclude modest acute-to-late associations. With 29 events and a minimum detectable OR of approximately 4.1 for the primary acute-to-late comparison, clinically meaningful associations cannot be excluded, and the absence of statistically significant findings should not be interpreted as proof that acute and late toxicity are unrelated. Second, allocation to radiotherapy technique was nonrandomized and determined by treating consultant discretion, taking into account the greater planning time, logistical complexity, and cost of IMRT. Although baseline characteristics were balanced, residual confounding arising from clinician preference or socioeconomic factors cannot be excluded, and the findings may not be generalizable beyond this single-institution cohort.

Third, late toxicity was assessed clinically and was not blinded to treatment allocation. No interobserver reliability data, objective salivary flow measurements (sialometry), or patient-reported outcome measures were available, leaving the possibility of differential misclassification, particularly for clinician-assessed xerostomia. Fourth, follow-up was limited to 12 months, whereas late xerostomia may continue to evolve and partial salivary recovery after IMRT may occur over two years or longer. Consequently, the observed differences at 12 months may not fully reflect long-term outcomes.

Fifth, the regression models did not adjust for tumor stage, primary tumor subsite, or delivered chemotherapy dose. The primary subsite is particularly relevant because laryngeal primaries generally receive lower parotid doses irrespective of radiotherapy technique. Although subsite distribution did not differ significantly between treatment arms (p = 0.340), laryngeal tumors were numerically more frequent in the 3D-CRT group (12 vs 7), representing a potential source of residual confounding in the dosimetric analyses. Sixth, this cohort overlaps two previously published reports, although the overlap, rationale for the secondary analysis, and the novel patient-level linkage, dosimetric analyses, and regression modeling are fully disclosed in the manuscript and under Additional Information.

Seventh, acute mucositis was analyzed using the prespecified grade 3 threshold in the primary regression models, whereas the only nominally significant univariate association was observed using ordinal Spearman correlation. Because a full ordinal regression analysis was not performed, this isolated univariate finding (ρ = 0.23, p = 0.042) should be interpreted cautiously, particularly as it disappeared after multivariable adjustment. Eighth, the dichotomized parotid-dose analysis should also be interpreted cautiously because, as shown in Table 5, the dose strata closely mirror treatment technique. Nearly all patients receiving a lower parotid dose <26 Gy were treated with IMRT, whereas almost all patients receiving ≥26 Gy were treated with 3D-CRT. Consequently, this analysis serves primarily as a mechanistic illustration of the technique effect rather than an independent line of evidence confirming the association between parotid dose and late xerostomia.

Finally, both multivariable regression models failed their overall likelihood-ratio tests and demonstrated only modest discrimination. Accordingly, these regression analyses should be regarded as exploratory and hypothesis-generating rather than confirmatory.

## Conclusions

In patients treated with definitive chemoradiotherapy for locally advanced HNSCC, we found no evidence that the severity of acute radiation toxicity predicts subsequent late xerostomia, dysphagia, or fibrosis. However, the cohort was too small to exclude moderate associations, and these findings represent a failure to demonstrate an association rather than evidence of true dissociation. The single apparent acute-to-late association was not significant after adjustment for treatment technique, suggesting confounding by technique. Late xerostomia was more closely associated with radiotherapy technique and mean parotid dose; patients receiving a lower parotid mean dose below the prespecified 26-Gy threshold experienced less than half the rate of grade ≥2 xerostomia, although these dose-based and regression estimates remain exploratory. Consistent with the distinct radiobiology of early- and late-responding tissues, acute reaction severity should not be used in isolation to stratify survivors for late-toxicity risk. Delivered parotid dose and treatment technique provide more clinically relevant indicators of late salivary morbidity. Wider access to IMRT and systematic attention to parotid dose remain important modifiable strategies for reducing late salivary toxicity in this population.
